# Magnesium chloride-calcium carbonate treatment in a pregnant patient with severe Darier disease

**DOI:** 10.1016/j.jdcr.2023.11.016

**Published:** 2023-11-30

**Authors:** Avalon Yi, Jiasen Wang, Deeptej Singh, Nikifor K. Konstantinov

**Affiliations:** Department of Dermatology, University of New Mexico, Albuquerque, New Mexico

**Keywords:** Darier disease, magnesium chloride-calcium carbonate, pregnancy

## Introduction

Darier disease is a rare autosomal dominant genodermatosis resulting from a mutation in ATP2A2 gene encoding calcium (Ca^2+^) ATPase pumps in the endoplasmic reticulum (SERCA2). This mutation leads to Ca^2+^ depletion resulting in acantholysis.^1^ This disease is characterized by keratotic and crusted papules and plaques in a seborrheic distribution, as well as palmoplantar papules, nail changes and whitish oral mucosal papules.^1^ Histology typically shows acantholytic dyskeratosis with suprabasilar clefting. Exacerbating factors include lithium carbonate, UV light, perspiration, and heat.^1^ Flares can present with pruritus, malodor or secondary infections, most commonly *Staphylococcus aureus*, human papilloma virus, and herpes simplex virus.^1^ Treatment includes topicals such as, retinoids, corticosteroids, calcineurin inhibitors, 5-FU, antibiotics/antifungals, as well as systemic therapies such as isotretinoin and cyclosporine.^2^ Other therapies include laser and surgical treatment.^2^

Flares in association with menstruation and pregnancy have been reported but are thought to not be as common because high estrogen states make Darier less active.^3^^,^^4^ For flares in pregnancy there are few safe treatment options. We report a case of a 29-year-old woman with Darier disease, who presented with a severe flare of her disease during pregnancy. She was successfully treated with an oral magnesium chloride-calcium carbonate supplement (MgCl_2_-CaCO_3_), a treatment which has only been reported once in the literature in a nonpregnant patient.^5^

## Case report

**Fig 1:**
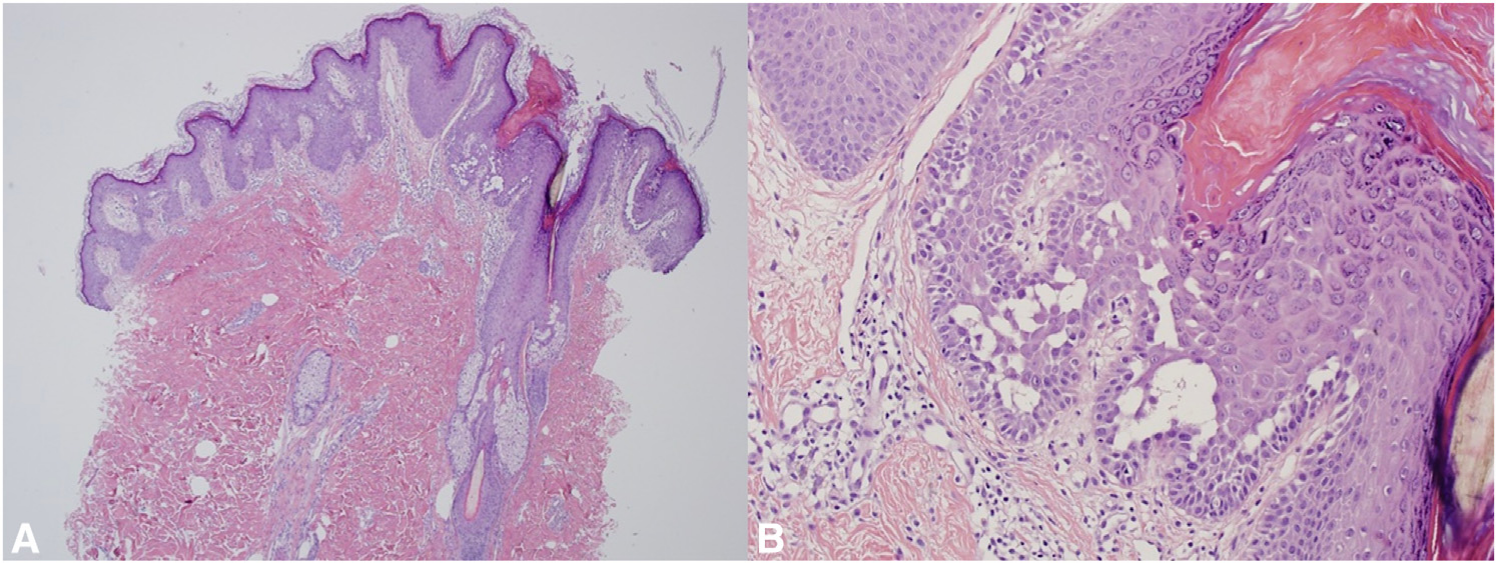
**A, B,** Punch biopsy of left axilla showing mild epidermal acanthosis with papillomatosis. On higher magnification suprabasilar acantholysis with dyskeratosis and formation of corp ronds and grains.

**Fig 2:**
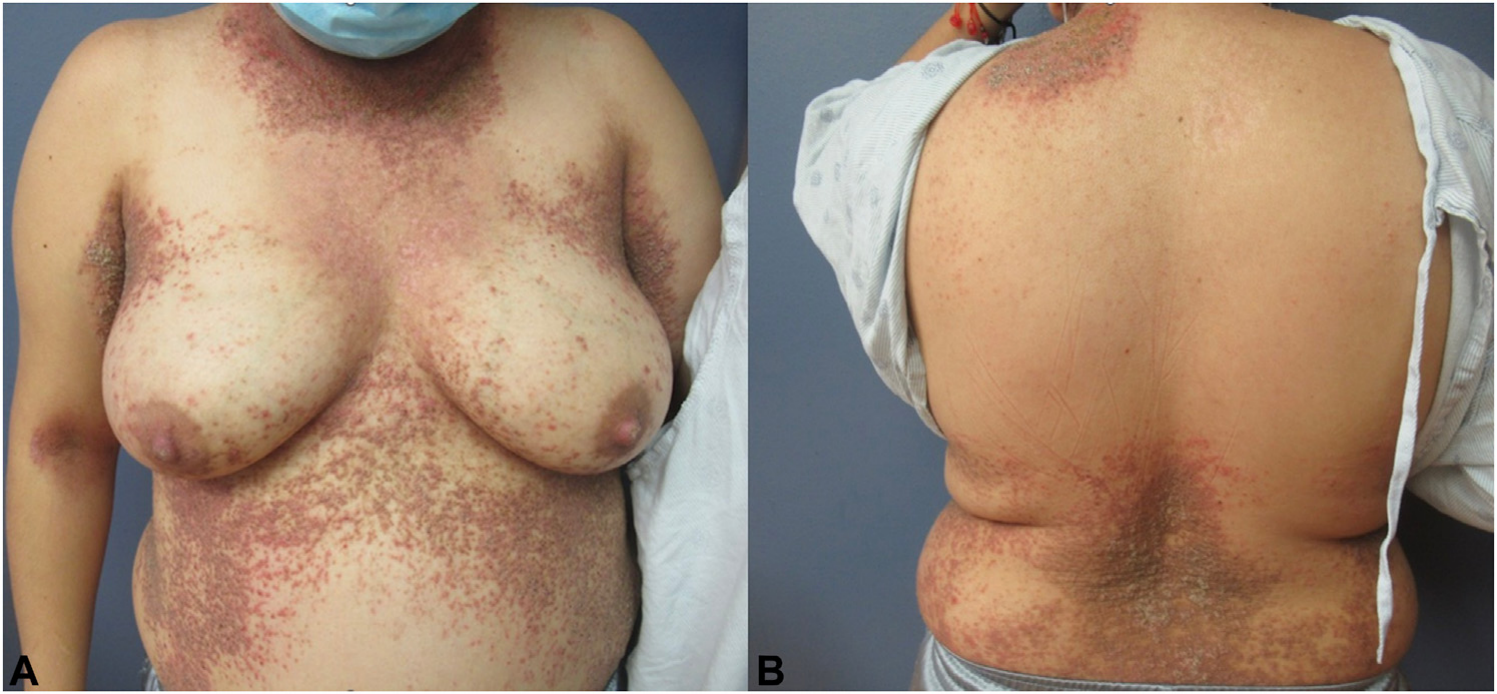
**A, B,** Presentation at initial visit pretreatment.

A 29-year-old female at approximately 6 weeks gestation with a history of Darier disease, diagnosed at age 14 years and confirmed via biopsy (Fig 1), presented with generalized cutaneous eruption consistent with her prior flares. On examination she was found to have bright red-pink eroded plaques with greasy yellow-brown crust with similar appearing satellite lesions on the central chest, lower back, flanks, neck, axillae, and flexural elbows (Fig 2). Culture swabs performed grew *S aureus* and herpes simplex virus 1. She was treated with cephalexin, valacyclovir, and topical corticosteroids (triamcinolone 0.1% and fluocinonide 0.05% ointments). During her pregnancy, she had 3 separate 7-day courses of cephalexin 500 mg tablets taken 4-times per day, due to repeat positive bacterial culture swabs (*S aureus* and *Staphylococcus agalactaie*). Once a day chlorhexidine gluconate 4% wash application was recommended on eroded areas on her skin. The patient had repeat viral swabs performed throughout her pregnancy which were negative for herpes simplex virus 1/2 and varicella-zoster virus and she did not require suppressive dosing after her initial 10-day course of valacyclovir.

**Fig 3:**
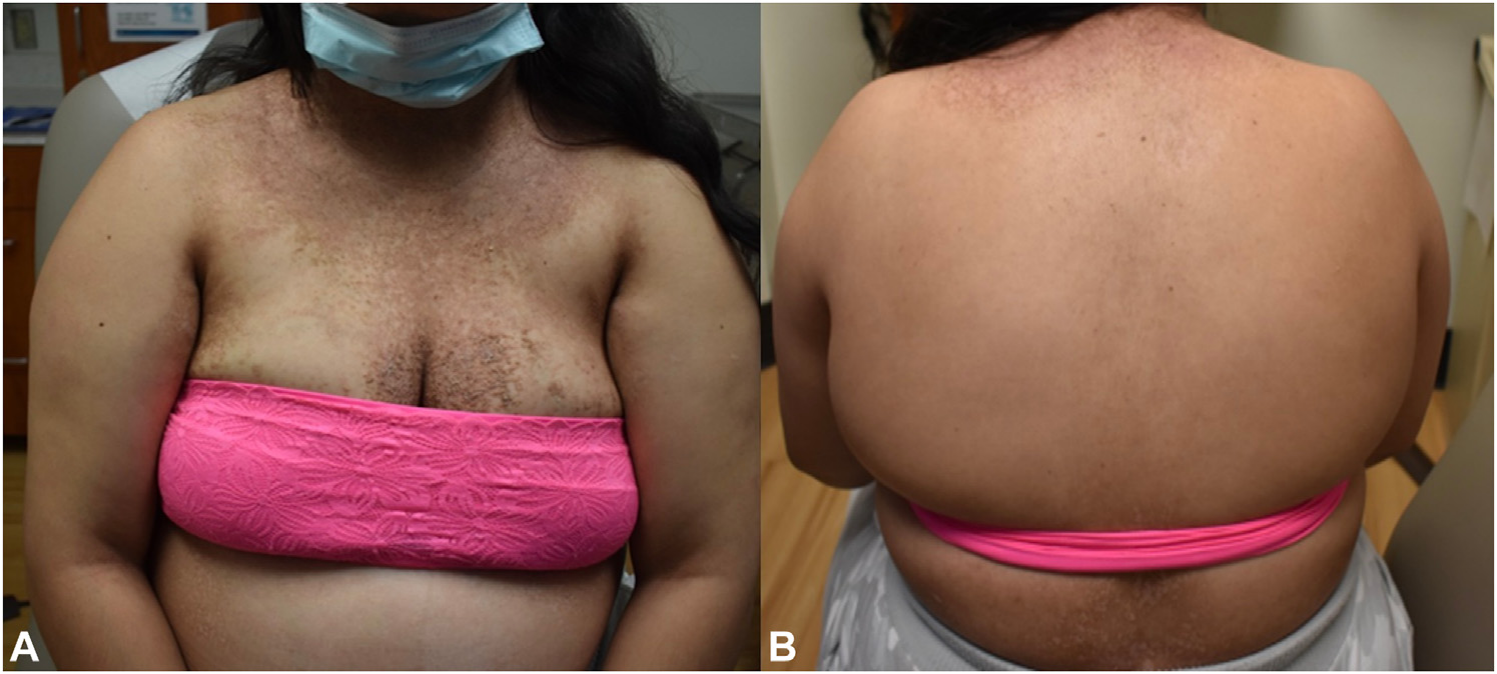
**A, B,** Presentation after 1 month of MgCl_2_ treatment.

However, after 1 month she had not experienced any improvement on this treatment regimen. The patient was started on MgCl_2_-CaCO_3_ 71.5 mg-119 mg 2 tablets twice daily in consultation with maternal fetal medicine who believed that was a safe treatment during her pregnancy. After 1 month of MgCl_2_-CaCO_3_ monotherapy, the patient experienced significant improvement in the lesions on her trunk and arms (Fig 3). The patient had a healthy and normal spontaneous vaginal delivery at 38 weeks of gestation. The patient has been seen in clinical follow-up postpartum and only has mild disease activity while she continues to take MgCl_2_-CaCO_3_ daily with occasional topical steroid application.

## Discussion

Darier disease flares have been reported in pregnancy,^6^^,^^7^ however there is also a hypothesis that Darier is hormonally dependent and less active in states of estrogen excess such as pregnancy.^8^ Safe and effective treatment options for Darier flares during pregnancy are limited. This case highlights treatment with MgCl_2_-CaCO_3_ 71.5 mg-119 mg 2 tablets twice daily, which may be a safe alternative therapy in patients who have recalcitrant disease or who have contraindications to other standard therapies. In Darier disease, the endoplasmic reticulum calcium stores are depleted. It is proposed that high magnesium levels lead to inhibition of Ca^2+^ ATPases restoring calcium stores that way processes requiring calcium, such as apoptosis and creation of desmosomes, can occur.^5^ The efficacy of MgCl_2_ in Darier disease has been reported once previously in an 11-year-old boy with recalcitrant disease, who experienced significant improvement after 1 month of treatment with this supplement.^5^ The MgCl_2_-CaCO_3_ was utilized instead of MgCl_2_ reported in literature because it was the only medication option in our hospital’s formulary.

Pregnancy may limit the treatment options in patients with Darier disease due to teratogenic effects of therapy, or lack of information regarding the safety profile of various treatments. Severe Darier disease is often treated with systemic retinoids as first-line therapy, which are contraindicated in pregnancy. Topical retinoids are also avoided in pregnancy. Cyclosporine, which can be used in severe Darier disease as a second-line agent, may be associated with premature delivery and low birthweight and use in pregnancy needs to be carefully considered by the treating physician.^6^ Given that infections are frequent triggers for flares of Darier disease, culture swabs for bacteria and herpetic infections should be done and treated appropriately. In this case the patient was not on herpes virus suppressive therapy, due to repeat negative viral cultures and lack of genital lesions. It is important to closely monitor patients for herpes infections during pregnancy and consider suppressive therapy in patients who develop recurrent infections, or presence of genital herpes from 36th week of gestation, as the consequences of neonatal herpes infection may be severe, and a cesarean-section may be required.

In the literature, there are cases of Darier flares in pregnancy reported however none of which tried MgCl_2_-CaCO_3_ as part of the treatment regimen and only one mentioning treatment course.^7^, ^8^, ^9^ In the reported case, bacterial super infections were treated appropriately and topical hydrocortisone and clobetasol propionate were used on the lesions with improvement.^9^ With limited choices in treating severe flares during pregnancy and the success seen in our case, MgCl_2_-CaCO_3_ 2 tablets twice daily, or MgCl_2_ 300 mg daily, may be a safe and efficacious treatment option for this patient population.

## Conflicts of interest

None disclosed.
